# The Neonatal Fc Receptor (FcRn): A Misnomer?

**DOI:** 10.3389/fimmu.2019.01540

**Published:** 2019-07-10

**Authors:** Michal Pyzik, Kine M. K. Sand, Jonathan J. Hubbard, Jan Terje Andersen, Inger Sandlie, Richard S. Blumberg

**Affiliations:** ^1^Division of Gastroenterology, Hepatology and Endoscopy, Department of Medicine, Harvard Medical School, Brigham and Women's Hospital, Boston, MA, United States; ^2^Department of Biosciences, University of Oslo, Oslo, Norway; ^3^Division of Gastroenterology, Hepatology and Nutrition, Department of Pediatrics, Harvard Medical School, Boston Children's Hospital, Boston, MA, United States; ^4^Department of Immunology, Oslo University Hospital Rikshospitalet, Oslo, Norway; ^5^Department of Pharmacology, Institute of Clinical Medicine, University of Oslo and Oslo University Hospital, Oslo, Norway; ^6^Harvard Digestive Diseases Center, Boston, MA, United States

**Keywords:** IgG, IgG immune complex (IgG-IC), albumin (ALB), FcRn, immunity, therapeutic

## Abstract

Antibodies are essential components of an adaptive immune response. Immunoglobulin G (IgG) is the most common type of antibody found in circulation and extracellular fluids. Although IgG alone can directly protect the body from infection through the activities of its antigen binding region, the majority of IgG immune functions are mediated via proteins and receptors expressed by specialized cell subsets that bind to the fragment crystallizable (Fc) region of IgG. Fc gamma (γ) receptors (FcγR) belong to a broad family of proteins that presently include classical membrane-bound surface receptors as well as atypical intracellular receptors and cytoplasmic glycoproteins. Among the atypical FcγRs, the neonatal Fc receptor (FcRn) has increasingly gained notoriety given its intimate influence on IgG biology and its ability to also bind to albumin. FcRn functions as a recycling or transcytosis receptor that is responsible for maintaining IgG and albumin in the circulation, and bidirectionally transporting these two ligands across polarized cellular barriers. More recently, it has been appreciated that FcRn acts as an immune receptor by interacting with and facilitating antigen presentation of peptides derived from IgG immune complexes (IC). Here we review FcRn biology and focus on newer advances including how emerging FcRn-targeted therapies may affect the immune responses to IgG and IgG IC.

## Introduction

It was F. W. Rogers Brambell who first proposed the idea of a fragment crystallizable (Fc) receptor system for Immunoglobulin G (IgG) after investigating the passage of maternal antibodies to fetuses and neonates (1). However, the identity of the specific receptor mediating this transfer, the neonatal Fc receptor (FcRn), remained unknown for nearly 30 more years (2) by which time other Fc gamma (γ) receptors (FcγR) had been identified (3–7).

As FcRn was structurally unique and not considered to be directly involved in immune responses, it was categorized as a non-classical FcγR that differs from the classical family members (Box 1) in several aspects (10). FcRn is distinctively a beta (β)-2-microglobulin (β_2_m) associated protein that is structurally related to the major histocompatibility class I (MHC-I) family, yet it is unable to present antigenic peptides to T cells (11). Further, FcRn has a quasi-ubiquitous expression pattern, possesses a predominantly intracellular localization, is monomorphic, and binds another, structurally and functionally unrelated protein to IgG, namely albumin (12). While the subtypes of IgG are fundamental in immune responses, albumin functions as a carrier protein in addition to being an important regulator of oncotic blood pressure (13). Despite these differences, IgG and albumin are the two most abundant serum proteins that possess a long serum half-life owing to their interaction with FcRn, which rescues them from intracellular degradation through a cellular recycling mechanism. Another of FcRn's functions is to transport IgG from mother to offspring thereby providing to the naïve and immature immune system of the newborn the experience and protection developed in the adult progenitor. This process is developmentally regulated in that it occurs antenally in rodents and humans through the inverted yolk sac or placenta, respectively, but uniquely continues at significant levels in the early post-natal life of rodents due to the high levels of FcRn expression in the intestinal epithelium. This functional expression of FcRn and its ability to transcytose IgG is not limited to the newborn but persists throughout life and permits the targeted delivery of IgG to sites where the presence of this type of antibody reinforces immunity, a process widely exploited by IgG-based therapeutics. Finally, the functions of FcRn are differentially determined by whether IgG is a single molecule, and thus monomeric, or present as an immune complex (IC). In the latter case, FcRn has been shown to critically regulate the innate immune responses as well as processing and presentation of antigens contained within IgG IC.

Box 1 **Classical FcγRs.**

Protein family of Fc receptors for IgG (FcγRs) which are broadly expressed by cells of hematopoietic origin. Can be divided into inhibitory (FcγRIIB) and activating receptors (FcγRI, FcγRIIA, FcγRIIC, FcγRIIIA, and FcγRIIIB). Through binding of IgG via the Fc portion, FcγRs are essential for regulating responses to infections and controlling inflammation (8, 9).

Here we review the versatile functions of FcRn in relation to albumin, monomeric IgG and IgG IC at different body sites. These observations have led to the emergence of protein-based therapeutics designed to harness, and in some cases, target FcRn function to promote the delivery of these therapies across mucosal barriers, increase their circulating half-life, or to treat IgG and IgG IC mediated diseases.

## FcRn Structure and Binding of Ligands

**Figure 1:**
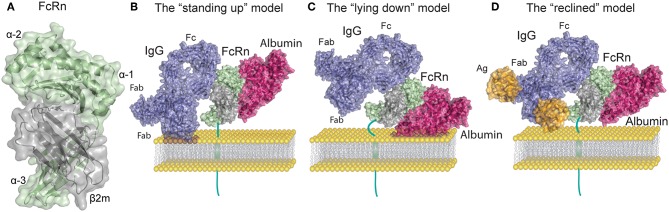
FcRn structure and ligand binding. **(A)** Human FcRn heavy chain (green) is non-covalently associated with beta-2 microglobulin (β_2_m, gray). **(B)** Topological representation of membrane associated FcRn in the standing up position. Bound albumin (magenta) and a monomeric IgG1 (blue) are modeled onto the structure of FcRn in complex with the Fc part of IgG^MST^ and albumin. In this orientation possible clashes may occur between the Fab arms of the IgG and the membrane. For simplicity the figures depict stoichiometric FcRn and IgG ratio of one to one. **(C)** Topological representation of membrane associated FcRn in the lying down position. This orientation accommodates Fab arms of IgG yet it potentially confines the albumin binding site. **(D)** Topological representation of membrane associated FcRn bound to albumin and IgG in complex with a small antigen [guinea fowl lysozyme (orange)]. The reclined orientation of FcRn on the surface of the endosomal membrane may most proficiently accommodate both ligands. Binding of large IgG IC (not depicted) might impose a lying down FcRn position. The figures were made using Adobe Illustrator, PyMol and the crystal structure data from: FcRn in complex with the Fc part of IgG^MST^ and albumin (23), a full-length human IgG1 antibody (24), and guinea fowl lysozyme (25), PDB IDs 4N0U, 1HZH and 1FBI respectively.

As an atypical FcγR, FcRn is structurally related to MHC-I molecules with a 40 kDa alpha (α) heavy chain that non-covalently associates with the 12 kDa light chain β_2_m (14–16). The FcRn heavy chain consists of three extracellular domains (α1, α2, and α3), a transmembrane domain and a cytoplasmic tail of 44 amino acids (16). Since the first crystal structure of FcRn was solved by Burmeister and colleagues (14, 17), several other published crystal structures have shown that the α1 and α2 domains form a platform of eight antiparallel β-strands with two α-helices on top while β_2_m is non-covalently associated with the α heavy chain (14, 18–22) (Figure 1A). Given its high similarity to MHC-I, FcRn was initially believed to present peptides (26), however the peptide binding groove of FcRn was subsequently found to be occluded (14). Instead this unusual FcγR binds with high affinity to IgG and albumin through non-overlapping sites at mildly acidic pH of 5.0–6.5 and exhibits no detectable binding to most of these ligands at neutral pH (Figure 1B), the exceptions being mouse IgG2b and some human IgG3 allotypes that display weak binding at neutral pH to mFcRn and hFcRn, respectively (27, 28).

FcRn interaction with the Fc portion of IgG occurs at the CH2 and CH3 domain interface, and involves the IgG Fc residue Ile253, and two central histidines: His310 and His435. Due to their pKa, the histidine residues become protonated at pH ~6 which allows for interaction with the FcRn residues Glu115 and Asp130 (Figures 1A,B). As the pH increases above 6, histidine protonation is gradually lost which explains the pH dependence of the interaction (23, 29, 30). In addition to the heavy chain interactions, β_2_m also forms contacts with IgG through the Ile1 residue (31). Mutating the IgG residues Ile253, His310, and His435 (IHH) leads to complete abrogation of FcRn binding at pH 6, which explains the reduced transcytosis and recycling of this mutated variant (32, 33). The FcRn binding site on IgG is distinct and distant from the binding site for classical FcγR which requires the glycosylation at the Asn297 residue of the Fc region of IgG (34).

Given that IgG is a homodimeric molecule, and contains two Fc domains, FcRn-IgG interactions have been proposed to occur with a stoichiometry of two FcRn molecules per one IgG (2:1). Indeed, an FcRn dimer was observed in crystals of the apo-FcRn (14), and in an FcRn-Fc complex (17, 35). Initially, the two binding sites on IgG for FcRn were not considered to be equivalent (35–37). Further studies with heterodimeric IgG, whereby only one of the sides of the Fc region was able to bind FcRn, showed reduced transepithelial transport in a model cell line (38). More recently it was shown that FcRn binds with equal affinity to each of the homodimeric wild-type (*WT*) IgG (39), but that the avidity effect resulting from the 2:1 complex formation was important for half-life extension (39). These results suggest that functional interaction of FcRn with monomeric IgG occurs with a 2:1 stoichiometry.

Recent work has indicated that in addition to the core Fc binding site on IgG, the fragment antigen binding (Fab) arms are also involved in FcRn binding (40–43). This was first suggested by experiments where antibodies with identical Fc but different Fab regions showed different affinity for FcRn and circulating half-life (40, 41). Accordingly, it was noted that the charge distribution of the Fab region, and the isoelectric point of the IgG itself can affect the dissociation from FcRn at physiological pH (41). As a result, a decrease in the rate of IgG dissociation from FcRn at physiological pH caused in faster *in vivo* clearance (41, 42, 44). Investigation of the FcRn IgG binding by the hydrogen deuterium exchange method has suggested a two-pronged interaction, involving direct interfaces between not only the IgG Fc region but also the Fab regions with FcRn (43). Nevertheless, surface plasmon resonance (SPR) studies with immobilized receptor could not detect differences in FcRn binding kinetics for IgG variants with different variable domains and different isoelectric points (39). Therefore, although it is incontestable that Fab regions can affect IgG binding to FcRn, the details of this involvement remain enigmatic.

Compared to IgG, binding of FcRn to albumin involves a larger surface area of the receptor, which is also more hydrophobic in nature than the IgG binding surface (23, 45, 46). Although this binding site for albumin on FcRn is located on the opposite side relative to that of IgG, it also relies on key histidine residues that bestow pH dependency to albumin-FcRn interactions (23, 47, 48) (Figure 1B). Albumin is a globular transport protein consisting of three structurally similar and highly flexible domains (49). Domain I (DI) and Domain III (DIII) are involved in its interaction with FcRn (Figure 1B). The main FcRn binding site consists of two hydrophobic pockets in albumin DIIIA and DIIIB that allow for binding of two FcRn tryptophan residues (Trp59 and Trp53) (23, 45, 50). Human albumin DI interacts with FcRn via two exposed loops that modulate FcRn binding (50, 51). However, similar participation of DI has not been observed for murine albumin, as this domain displays negligible contacts with mouse FcRn (52). Further, His166 of human FcRn (corresponding to His168 of mouse FcRn) is crucial for this interaction, and alanine substitution of this residue abolishes albumin binding (48). This occurs because at mildly acidic pH, His166 forms intramolecular hydrogen bonds that constrain the position of the loop containing Trp59 and Trp53, which are needed for albumin binding (23, 45, 48, 51). Several other histidine residues (His464, His510, His535) and Lys500 in albumin are also important for the interaction, and mutating any of these, reduces its binding to FcRn (47). Unavoidably, FcRn contact sites on albumin are also the binding sites for albumin cargo such as fatty acids, thyroxine, and drugs, as has been reviewed in (53). Thus, albumin molecules carrying long chain fatty acids exhibit reduced binding to FcRn (45, 54). These observations suggest that failure of albumin binding to FcRn may be used to optimize albumin-cargo delivery into cells due to decreased albumin recycling, in addition to its detrimental effects on the half-life of cargo-bound albumin. The diversity of albumin interactions with its cargo adds complexity to the mechanisms underlying albumin half-life and suggests a cellular mechanism for how albumin loaded compounds are delivered to cells.

*In vitro* protein-protein interactions and crystallographic studies of FcRn bound to albumin and IgG Fc have both shown that the receptor can engage its two ligands simultaneously, which is in line with the fact that the binding sites are non-overlapping (23, 55) (Figure 1B). Nonetheless, studies that only rely on soluble FcRn forms, without assessing surface immobilized receptor binding, as well as *in vitro* cellular assays or *in vivo* studies should be taken with caution. Early crystallographic data from the Bjorkman laboratory has put forward two putative models of FcRn Fc binding, wherein FcRn assumes either a perpendicular (“standing-up”) or supine (“lying-down”) position relative to the membrane (11, 17) (Figures 1B,C). Due to expected collisions between the Fab arms and the membrane surface inherent in the former model, it was considered less functional. It is interesting to note that mouse MHC-I molecules have been shown to exist on the surface of cell membranes in the lying down position, supporting the latter model (56). However, such an orientation of FcRn might render the albumin binding site of FcRn difficult to access. A recent study by Booth et al. highlighted the physiological relevance of membrane-bound FcRn orientation, and illustrated that upon binding to monomeric IgG, FcRn may direct the antibody into a T-shaped conformation to allow for minimal steric hindrance with the membrane bilayer (57). Such a scenario is enabled by the marked flexibility of the Fab domains of IgG (58, 59), which can assume many different positions relative to the Fc (60–63), and also modulate FcRn binding (40, 41). Given these spacial restrictions an intermediate, “reclined” position of membrane-bound FcRn may be more likely to accommodate both IgG and albumin binding when compared to the “standing up” or the “lying down” models (Figures 1B–D). All these factors are particularly important when considering FcRn interactions with IgG IC, where Fab arms bound to antigen forming large IC may encounter even stronger steric effects.

When looking at the available binding affinities of FcRn to its ligands, diverse quantitative measurements have been published for these interactions (11, 28, 34, 35, 48–50). Consequently, to assess these reports one must consider the different experimental designs, natures of assayed reagents, as well as variability within the ligands themselves. SPR studies at acidic pH have reported that the K_D_ value for the human FcRn-albumin interaction (~1 μM) is around 7-fold higher when the receptor is immobilized as compared to a design utilizing albumin immobilization (~0.2 μM) (50, 55, 64). For the human IgG-FcRn interactions at acidic pH, the SPR-derived K_D_ is even more sensitive to the experimental setup, as the affinities reported when IgG1 is immobilized vary from ~0.2–2.3 μM, whereas the values are in the nanomolar range when FcRn is immobilized (~10–100 nM) (39, 65–68). The latter values are likely affected by the avidity effect from IgG's two binding sites for FcRn. One recent study compared albumin and IgG binding to FcRn in solution using microscale thermophoresis, which gave a K_D_ of 0.9 and 0.5 μM for albumin and IgG, respectively (69). Furthermore, diversity in binding affinities is also seen between FcRn and IgG or albumin from different species or across species (27, 68, 70). Thus, caution should be taken with the extrapolation of animal models to hFcRn and IgG or albumin interactions and vice versa.

## Cellular Transport Mechanisms

The pH-dependent ligand binding is crucial for all FcRn functions: including recycling and transcytosis, which allow FcRn to salvage its ligands from intracellular degradation pathways, to transport them across cell layers, and to potentiate efficient immune responses to antigen in the case of IgG IC.

**Figure 2:**
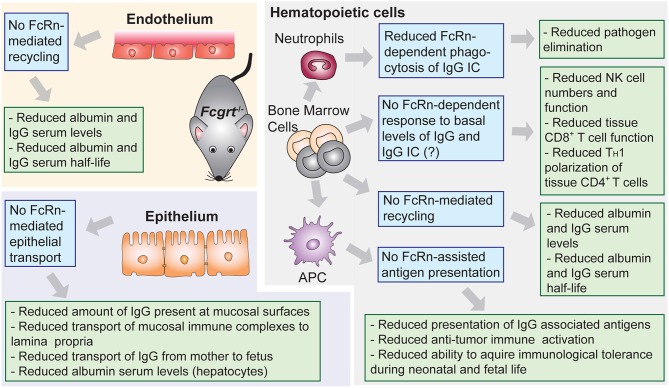
Lessons from conventional and conditional FcRn deficient mouse models. Phenotypic observations reported in mice deficient for FcRn (Fcgrt^−/−^). Blue boxes denote FcRn-affected function and green boxes denote pathophysiological consequences.

The understanding of the central role of FcRn as a homeostatic regulator of circulating levels of IgG and albumin derives from studies in mice with conventional (*Fcgrt*^−/−^) (71) or conditional (*Fcgrt*^fl/fl^) (Box 2) (73) deletion of the FcRn heavy chain gene (Figure 2), although as a correlate, the β_2_m light chain deficient mice (B*2m*^−/−^) were also initially utilized (46, 74–78). Importantly, no human case of FcRn heavy chain deficiency has ever been reported, and the only clinical data available regarding the effects of FcRn deficiency in humans, comes from investigations of a rare human syndrome called familial hypercatabolic hypoproteinemia (79, 80). Affected individuals carry a mutation in β_2_m that prevents cellular expression of β_2_m protein and its associated heavy chains, including FcRn (81, 82). Normally the half-life of human IgG and albumin is around 19–21 days, while most other serum proteins, such as IgA, have half-life of ~5–7 days at the longest (83). In the case of familial hypercatabolic hypoproteinemia two described patients had significant reductions in both IgG and albumin serum levels, with IgG and albumin half-lives of ~3 and ~6 days, respectively (80). In *WT* mice, the half-life of albumin and IgG was observed to be ~39 and 95 h, respectively, compared to 25 h for IgA (46). The deletion of murine FcRn heavy chain, resulted in significant reduction of IgG and albumin half-life to ~22 h (46), with concomitant decrease in circulating levels of IgG and albumin from ~1.5 to 0.5 mg/ml and from ~45 to 20 mg/ml, respectively (46, 66, 71, 75, 77, 78).

Box 2 **Cre–lox recombination.**

A site-specific recombinase technology that allows DNA modification targeted to a specific tissue or cell type, or to be triggered by a specific external stimulus. Relies on the DNA recombinase Cre and its recognition (loxP) sites. For conditional mutagenesis a target gene is modified by the insertion of two loxP sites that enables excision of the flanked (floxed) gene segment by Cre-mediated recombination. The floxed strain can further be crossed with a Cre transgenic line resulting in target gene inactivation *in vivo* within the expression domain of Cre (72).

The mechanism underlying these FcRn-mediated effects on IgG and albumin half-life is the pH-dependent diversion of both ligands from intracellular degradation pathways. This FcRn-dependent rescue of IgG from lysosomal degradation is a saturable process, such that administration of high doses of IgG (but not IgM, IgA or albumin) accelerates the clearance of endogenous IgG (84). Albumin injected into hypoalbuminemic individuals shows a half-life 4–5-fold longer than normal, which is in line with the rate of albumin salvage being also sensitive to FcRn saturation and expression levels (85–87).

The cell biological basis for intracellular recycling of IgG has been studied extensively by Ward et al., using mostly endothelial cell lines transfected with a fluorescently tagged FcRn (88–93). In these cells FcRn is known to localize intracellularly mainly to early endosomes positive for Rab5, EEA1 and recycling endosomes positive for Rab4 and Rab11a (Box 3) (90, 92). IgG is thought to enter endothelial cells non-specifically in pinocytocytic vesicles and subsequently bind to FcRn in EEA1-, Rab5-, Rab4-, and Rab11a-positive sorting endosomes characterized by pH of ~6. IgG-bound FcRn then separates from sorting endosomes to Rab4- and Rab11a-positive recycling endosomes. The IgG variant His435Ala, which does not bind to FcRn, is instead sorted to lysosomes (89, 93). Recycling of FcRn bound IgG proceeds through multiple types of exocytic processes, including the fusion of Rab11a-positive vesicles that contain both FcRn and IgG with the plasma membrane for rapid release, or the so-called “prolonged release,” where multiple pulses of IgG excretion can occur over a longer period of time (90). Intracellular trafficking-studies of IgG IC have shown that, whereas monomeric IgG and small IgG IC follow the recycling pathway, large IgG IC are mainly sorted to lysosomal compartments (96). This has been shown in human monocyte derived dendritic cells (DC), in which FcRn transports IgG IC to degradative compartments (LAMP1^+^) involved in antigen presentation (97). As the intracellular trafficking of FcRn has been mainly studied using IgG as a ligand, it is unknown whether albumin recycling is governed by the same principles. Further, intracellular sorting of albumin and IgG have not been directly compared in the same experimental system. Recent studies in a human endothelial cell line have however shown sorting of albumin to early endosomes positive for EEA1; the recycling of albumin was noted with variants having high FcRn affinity, and lysosmal sorting of albumin variants with low FcRn affinity (98, 99).

Box 3 **Rab proteins.**

Large protein family of small Ras-like GTPases that are regulators of vesicle trafficking in cells. They control vesicle budding, uncoating, fusion and membrane identity through recruitment of effector proteins (94, 95).

The FcRn transcytotic trafficking has mainly been studied using the model epithelial cell line from dogs, Madin-Darby Canine Kidney II cells (MDCK II). In this model, the cellular regulators of FcRn-IgG transcytosis differ from those involved in recycling, and FcRn mediated transcytosis in both directions requires both Myosin Vb and Rab25 (100). In addition, calmodulin, which can bind to the membrane proximal part of the cytoplasmic tail of FcRn in a calcium dependent fashion, is involved in this process (101). As is the case for recycling, endosomal acidification is also required for FcRn-mediated transcytosis (102–104). In MDCK II cells stably expressing human FcRn/β_2_m, the receptor localizes mainly to apical vesicular structures and has been shown to traffic more frequently to the basolateral membrane, a process which relies on the presence of tryptophan and leucine residues in the cytoplasmic tail of FcRn (105–107). Electron tomography studies using rat intestinal epithelial cells have shown that clathrin is associated with the endocytotic and exocytotic processes involving FcRn, which supports the notion that it is rapidly retrieved from the plasma membrane after exocytosis (108). In contrast, rat FcRn displays opposite polarity when expressed in MDCK II, trafficking predominantly in a basolateral-to-apical direction. Such distribution depends on differences in receptor glycosylation, as rodent FcRn has four glycosylation sites and human has only one (109). Furthermore, using rat inner medullary collecting duct cells, transcytosis of rat FcRn in the apical to basolateral direction was shown to require phosphorylation of a serine residue (Ser313) in the cytoplasmic tail, whereas transcytosis in the basolateral to apical direction did not (110). Thus, the ligand-sorting and transcytotic functions of FcRn are mediated by specific regions and residues of the cytoplasmic tail of FcRn, which may differ between species.

Given that FcRn is a mostly intracellular receptor with functions that depend on its trafficking in the recycling and transcytotic pathways, surprisingly few studies have focused on how FcRn's intracellular trafficking is regulated. This might vary according to cell type, nature of the specific ligand and its valence as well as its interplay with other receptors or regulators of intracellular trafficking.

## Functional Consequence of FcRn Expression in Epithelium

As non-classical MHC-I family members are characterized by unique and more restricted expression patterns than classical MHC-I molecules, it was initially surmised that FcRn, mediating transport of IgG from mother to offspring, was only present in placental and intestinal tissues during the fetal and neonate period. Since then, however, FcRn expression has been detected almost ubiquitously in diverse tissues throughout the body including epithelia, endothelia and cells of hematopoietic (HC) origin. FcRn epithelial expression has been shown in the intestines (enterocytes) (102, 111, 112), placenta (syncytiotrophoblasts) (113), kidney (podocytes and renal proximal tubular cells) (114), and liver (hepatocytes) (115).

### Intestinal FcRn

It was more than 40 years ago that Jones (116) and Rodewald (117) described age- and tissue-specific transfer of IgG in rodents. They illustrated that segments of the proximal jejunum but not ileum of 10–14-day old rats transported only IgG from the lumen to the circulation, which was non-detectable in 22-day old rats. Subsequently, the receptor responsible for this transport was isolated from the proximal small intestine of neonatal rats (2). Since then, studies in humans characterized FcRn expression at intestinal mucosal surfaces throughout life in both the small and large intestine, including villous and crypt enterocytes in addition to goblet cells and sub-populations of enteroendocrine cells (102, 111, 112, 118). In these cells, FcRn was located mainly intracellularly and on the apical membrane lining the gut lumen.

It is important to mention that in humans, little maternal IgG is transmitted to the neonatal circulation across the intestines, as most of humoral immune competency is assured by placental transfer. In contrast, FcRn-mediated uptake of IgG in rats and mice occurs both during the fetal and neonatal periods via transfer across the inverted yolk sac placenta and intestine, respectively. In cattle and pigs, the neonates rely entirely on postnatal uptake of colostral antibodies, mainly IgG, via intestinal epithelium for systemic humoral immune protection. These differences are also reflected in the levels of antibodies present in colostrum and milk, where IgG represents up to 3% of total antibody levels in humans as compared to 80% in cattle (119). Despite these species-specific differences, it is clear that FcRn consistently plays a central role in establishing humoral immunity in mammalian offspring.

**Figure 3:**
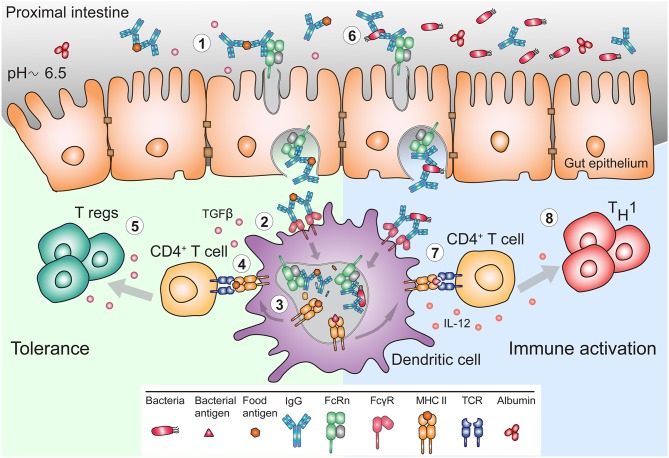
FcRn mediates bidirectional transport and immune response to IgG and IgG immune complexes in the gut. (1) The pH of the mucosal surface of the proximal intestine can be slightly acidic, such that FcRn can bind maternal IgG and IgG IC already at the cell surface, and transcytose these to the basolateral side. (2) APC such as DC, can bind and actively internalize IgG IC via FcγR. (3) FcRn in APC assists in antigen processing and delivery of the IgG IC to antigen loading compartments where peptides derived from these complexes can be loaded onto MHC II for presentation to CD4^+^ T cells. (4) In early life, presentation of antigen-derived peptide on MHC II in presence of other maternal milk-derived factors provides (5) tolerogenic environment to CD4^+^ T cells. In these instances, FcRn expression by APC is crucial for induction of CD4^+^Foxp3^+^ regulatory T cells (Treg). (6) In adulthood, during infection, pathogen derived antigens bound by lumenal IgG will be transported across mucosal membrane in an FcRn-dependent manner and (7) delivered to APC, which process and present antigens, (8) for subsequent activation of immune responses.

While the evolutionary fitness afforded by FcRn in early life is apparent, its utility in adults to justify life-long expression in the intestine is less well-understood. Experiments in murine model systems have demonstrated that circulating monomeric IgG can be delivered into the intestinal lumen of FcRn humanized mice but not of *Fcgrt*^−/−^ mice (120). Accordingly, IgG is present in mucosal secretions of the gastrointestinal, respiratory and genital tract where IgG antibodies together with IgA and IgM function together in host defense (121). However, while dimeric IgA and pentameric IgM is transcytosed unidirectionally via the polymeric immunoglobulin receptor, FcRn expressed in epithelial cells mediates transcytosis of IgG in both directions (105, 112, 120, 122, 123). Thus, FcRn in the intestines can deliver IgG into the lumen, and it also transcytoses monomeric IgG or IgG IC in reverse direction back into the lamina propria (Figure 3). This process ensures specific delivery of luminal antigens in the form of IgG IC to mucosal dendritic cells that can then regulate immune responses (120, 122). Indeed, the absence of FcRn results in greater susceptibility to mucosal infections with pathogens such as *Helicobacter pylori, Citrobacter rodentium*, or *Chlamydia muridarum* (122, 124, 125).

Although FcRn dependent transcytosis of IgG in the gut is well-established, the evidence for albumin transport has only recently been established. In initial studies, bovine serum albumin (BSA) conjugated to ferritin was not transported into the circulation of neonatal rats (117). On the other hand, Udall et al. showed that significant absorption of BSA occurred within first week of life in rabbits (126). More recently, investigations using MDCK II cells expressing human FcRn and β_2_m showed bidirectional albumin transcytosis (54). Epithelial transcytosis of albumin was also reported in another *in vitro* study using Caco-2 cells (127). Given that very little albumin is lost in the gastrointestinal tract, it is possible that any proximal transport of albumin into the intestinal lumen might be compensated for by FcRn-reuptake or alternately by reabsorption. Such mechanisms might explain the progressive increase in FcRn expression levels from duodenum to proximal colon (112), as well as the presence of cubilin in human small intestine (128) (Box 4), which would allow for receptor-mediated uptake of albumin similar to processes occurring in the proximal tubules of the kidney (130).

Box 4 **Cubilin and megalin.**

Cubilin is a large endocytic receptor responsible for intestinal absorption of the intrinsic factor vitamin B-12 complex, and renal tubule reabsorption of filtered plasma proteins including albumin, transferrin, vitamin D binding protein etc. Megalin (also known as LRP2) is another large endocytic receptor that belongs to a family of receptors with structural similarities to the low-density lipoprotein receptor (LDLR). Cubilin is a peripheral membrane protein that is dependent on megalin for efficient reabsorption in the kidney. Intestinal reabsorption of vitamin B-12 requires the protein amnionless, which is also needed for appropriate plasma membrane localization of cubilin (129).

As mentioned above, IgG IC are transported by hFcRn-expressing transgenic mouse gut epithelial cells in an inflammatory setting of *E. coli* infection (122). Notably, the original experiments of Rodewald utilized ferritin conjugated immunoglobulins, which are large protein complexes (117). Currently, studies in lactating female mice sensitized to different allergens during pregnancy have illustrated FcRn-mediated transport of IgG IC from breast milk across the gut epithelium (131–135). In this context, the transfer of antibody-antigen conjugates resulted in induction of tolerance to allergen in the offspring (131–133, 135). Recent reports have also supported the role of FcRn in intestinal transport of anti-IgE-IgG IC (136).

### Placenta

In line with FcRn function in transferring IgGs from mother to neonates across the gut epithelium in rats, observations from other species, notably humans and rabbits, have shown prenatal transport of IgG across the placenta or yolk sac, respectively (137). The species-specific fetal or neonate transfer of IgG has mainly been explained by placental anatomy differences across species and the level of placental invasiveness (138). For instance, in ruminant epitheliochorial placenta, six tissue layers (maternal capillary endothelium, maternal uterine connective tissue, uterine endometrium, trophoblast, embryonic connective tissue, and embryonic capillary endothelium) are interposed between the maternal and fetal circulations, while in the human hemochorial placenta three layers (trophoblast, embryonic connective tissue, and embryonic capillary endothelium) typically separate the two circulations. Thus, transport of IgG from mother to fetus in humans involves fewer cellular layers to traverse. Human FcRn has been found in both fetal endothelium and apically localized vesicles within the syncytiotrophoblasts that are in direct contact with maternal blood (113, 118, 139, 140).

IgG is the only antibody class that is transported across the placenta (141, 142), and this process is dependent on FcRn (66). Of the four IgG subclasses IgG1 and IgG4 are transported readily, whereas IgG2 and IgG3 show somewhat less efficient transplacental passage (28, 142, 143). In *ex vivo* human placenta transport studies, model IgG molecules disabled in FcRn binding did not cross to the fetal circulation (66, 144), while, conversely, an IgG variant with improved affinity for FcRn was transported more efficiently (145). Also, polarized human trophoblast-derived BeWo cells exhibited apical to basolateral IgG transcytosis and apical IgG recycling (146).

Classical FcγR (FcγRII and FcγRIII) have been detected in placenta and postulated to potentially participate in transplacental IgG transfer, whereas other studies could not find evidence for this (147–154). FcγRIIb2 is expressed in placental endothelial cells and FcγRIII in syncytiotrophoblasts (147–153). Pointing against involvement of FcγRs is the fact that an IgG3 variant with hinge-region deletions that prevents binding to all FcγR but retains FcRn binding was still transported to the fetus (154). Likewise, aglycosylated IgG variants that are unable to interact with FcγR, but bind FcRn, were transported in mice (155). Comparison of glycosylation patterns between fetal and maternal IgG showed that IgG transport was not glycosylation selective (143). In any case, the differential transport of IgG subclasses suggests that other factors in addition to FcRn may be involved in transplacental transport.

In rodents, a major anatomical difference is the presence of chorioallantoic placenta as well as second inverted yolk sac placenta, where IgG transport is thought to occur throughout the gestation. This is supported by detection of FcRn in yolk sac endoderm and its absence in mouse chorioallantoic placenta (152). The crucial role of FcRn in transfer of IgG was demonstrated in offspring from heterozygous FcRn deficient mice. FcRn deficient fetuses displayed negligible levels of IgG compared to FcRn-heterozygous or *WT* littermates (152). Similar and efficient transplacental transfer of Fc-fusion proteins (such as Factor VIII-Fc) have been observed in mice (156, 157).

Transport of albumin across the placenta does not seem to occur to the same extent as for IgG. In a study from 1964, pregnant women in the last trimester of pregnancy were injected with radio-labeled IgG or albumin (158). While the levels of labeled IgG were found to be higher in the offspring than in mother's circulation, the levels of labeled albumin were only about 15% of the amount detected in the mother. It is still unknown why albumin and IgG are transported differently, but involvement of other albumin receptors could be part of the explanation. For example, megalin and cubilin (Box 4) have been found to be expressed in the placenta (159–161), and it has been suggested that they might facilitate retrograde recycling of albumin back to the maternal circulation (159).

Whether IgG IC cross the placenta in an FcRn dependent fashion is also less studied. By comparing concentrations of tetanus antigen and anti-tetanus IgG in maternal and fetal blood, Malek et al. observed that the ratio of antigen to antibody in the fetal circulation closely fit the maternal levels, suggesting transfer of IgG IC (162). In addition, May et al. illustrated placental transfer of IgG IC consisting of IgG and *Plasmodium falciparum* merozoite surface protein 1 (MSP1) from women in malaria endemic areas (163). More specifically, MSP1 was regularly detected in cord blood complexed to an antibody, and using an *ex vivo* human placental model, MSP1 IgG IC transport from maternal to fetal circulation was observed. MSP1 alone or with plasma from non-immunized individuals was not transported (163). Recent reports have also illustrated FcRn-mediated transplacental transport of maternal IgE through interactions with anti-IgE-IgG (164). First, IC in the form of anti-IgE-IgG bound to IgE were transported across polarized MDCK II cells in an FcRn dependent manner, and most of the IgE present in cord blood sera was found in complex with IgG (164). These studies indicate that FcRn mediates transplacental passage of not only monomeric IgG but IgG IC as well.

Elucidating the mechanisms behind the transport of FcRn ligands across the placenta will be crucial to understand immune responses occurring at the materno-fetal interface. In addition, it may provide knowledge to develop precision treatments targeting the mother or the fetus without reciprocally affecting the other. For example, in multiple fetal alloimmune diseases, including fetal thrombocytopenias and rhesus disease, preventing the transmission of maternal autoimmunity to the fetus may be transformative (165).

### Kidney

Passage of proteins larger than 60–70 kDa into the urine is prevented by the charge- and size-selective filtration membrane in the glomeruli of the kidneys. Together with fenestrated endothelial cells, and the basement membrane, the kidney filtration barrier also consists of podocytes: large cells with foot processes that gate the basement membrane. Podocytes have been shown to express FcRn (114), and can transcytose IgG from the filtration membrane for delivery to the urinary filtrate (166). It is believed that this process serves two purposes: to clear IgG and IgG IC from the filtration membrane and to provide protective IgG to the urinary tract. Thus, *Fcgrt*^−/−^ mice show accumulation of IgG in the glomerular basement membrane which subsequently can lead to serum-induced nephritis (166). FcRn in the kidney is important also for albumin homeostasis, as mice lacking FcRn have reduced serum levels of albumin, which can be rescued by transplantation of an FcRn expressing kidney (167).

The proximal tubule epithelial cells line the inside of the proximal tubules and are involved in reabsorption of proteins from the filtrate. FcRn in these cells has been shown to be involved in reabsorption of albumin and potentially IgG (130, 168), and one study demonstrated that the proximal tubule epithelial cells were involved in albumin reabsorption using inducible podocyte-specific tagged albumin expression (130). The reuptake of albumin from the glomerular filtrate also depends on the cubilin-megalin receptor complex (Box 4) which specifically endocytoses albumin from the renal filtrate, and delivers it to intracellular compartments where FcRn operates (130, 169–172). The functional interaction of FcRn with the cubilin-megalin receptor complex is an important mechanism of synergy between surface and intracellular receptors that are specific for albumin (130). Further work should investigate the interdependence between these receptors and address their potential interactions at different anatomical sites where both are expressed, such as the placenta and the intestine (99).

### Liver

One of the important FcRn sites in the body is the liver (73, 118, 173). Indeed, the discovery of FcRn expression in adult rat hepatocytes was the first evidence that this receptor was expressed outside of the neonatal period (115). Since then, tissue expression of FcRn in humans, primates, rats, *WT* as well as humanized FcRn transgenic (TG) mice (174–176) has confirmed that the liver is a major site of FcRn expression, where its presence has been detected in liver endothelium, liver sinusoidal epithelial cells (LSEC), Kupffer cells, hepatocytes and perhaps biliary epithelium (118, 173, 177). Using the human liver hepatocellular carcinoma cell line, HepG2, D'Hooghe et al. illustrated that the majority of FcRn is distributed intracellularly mostly in the early, late or recycling endosomes, and to a lesser extent in the trans Golgi network or lysosomes (178). The remaining small fraction of FcRn was present on the cell surface and could be subdivided into two pools: one that underwent rapid endocytosis and the other that was endocytosis resistant. The functional significance of this FcRn expression pattern is unknown. Furthermore, studies with human FcRn/β_2_m^TG^ (*FCGRT*^TG^) mice have also illustrated that FcRn was distributed intracellularly in addition to being associated with the sinusoidal and canalicular hepatocyte surfaces (54).

The relative contribution of hepatic FcRn to IgG or albumin biology is still emerging. On one hand, the liver eliminates complex macromolecules from the circulation such as IgG IC, while on the other, it produces albumin. Analyzing IgG biodistribution data from mice, rat, monkey, and humans, Shah and Betts showed that the liver contained ~12% of the antibody attributed to the plasma compartment (179). Biliary excretion accounts for a very small amount of the eliminated IgG (54), and any hepatic IgG degradation that takes place likely occurs via intracellular catabolism in lysosomes. Studies in *WT* and FcRn deficient mice injected with antibodies labeled with either non-residualizing ^125^I- or residualizing ^111^I isotopes have illustrated a significant increase in IgG catabolism by the liver in the absence of FcRn (180, 181). Similarly, IgG antibodies that are unable to interact with FcRn are mainly catabolized in the liver, while *WT* antibodies are degraded mainly in the spleen, demonstrating that the liver possesses an important FcRn-mediated recycling capacity of monomeric IgG (182). Although hepatocytes were shown to efficiently recycle IgG Fc fusion proteins (183), absence of FcRn in hepatocytes did not significantly affect the circulating levels of IgG (54). Therefore, the specific cellular subset responsible for protection of monomeric IgG in the liver is still unknown. This is in contrast with small IgG IC that are eliminated efficiently from the circulation by the cells of the classical reticuloendothelial system (184, 185), mostly by the LSEC and also to some extent by Kupffer cells (186). This process relies on the expression of FcγRIIb, while the role of FcRn in LSEC has not been assessed (187).

**Figure 4:**
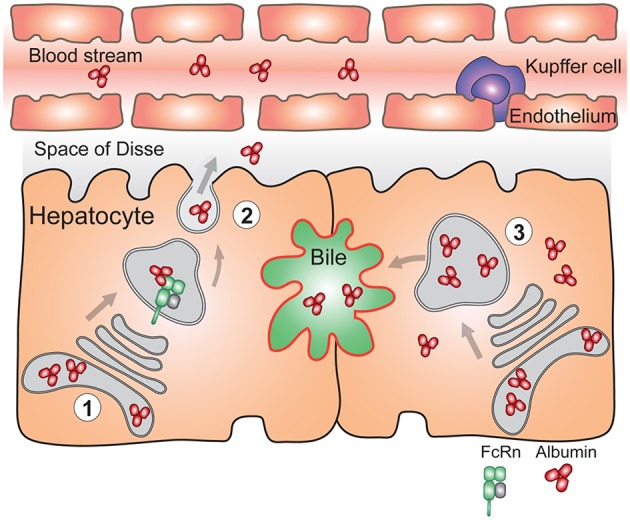
FcRn in the liver is essential for vectorial delivery of albumin into the blood stream. (1) Hepatocytes are polarized epithelial cells of which the apical side (red) faces the bile duct, and the basolateral side (black) faces the fenestrated sinusoidal endothelium. The sinusoidal endothelium is populated by liver specific macrophages called Kupffer cells. Albumin is produced solely by hepatocytes. (2) FcRn in hepatocytes is required for delivery of newly synthesized albumin to the basolateral side of the cells, and subsequent secretion of albumin to the blood stream (left) (3) Absence of FcRn expression in hepatocytes results in increased albumin levels in the bile, its intracellular accumulation and lower circulating albumin levels (right). For simplicity, FcRn-mediated albumin recycling in hepatocytes is not depicted.

As it pertains to the albumin homeostasis, both FcRn deficient humans (80, 81) and mice (46) are hypoalbuminemic. In *Fcgrt*^−/−^ mice, the hepatic albumin production rate is paradoxically increased by ~20% compared to normal mice, which is thought to represent a compensatory mechanism for the low circulating albumin levels (188). However, conditional deletion (Box 2) of FcRn in the liver which mainly affects hepatocytes (*Alb*^cre^*FcRn*^fl/fl^) resulted in inability to efficiently deliver albumin into the circulation. Thus, in the absence of FcRn, hepatocytes accumulated albumin intracellularly and biliary excretion of albumin significantly increased (54) (Figure 4). Using a polarized model cell line that co-expresses FcRn and albumin, it was shown that enhanced secretion of newly synthesized albumin occurred into the basolateral space modeling the bloodstream, rather than into the apical space which modeled the biliary ducts. Lack of FcRn resulted in mostly apical albumin secretion as well as intracellular accumulation (54). Thus, the presence of FcRn within hepatocytes mediates physiological albumin biodistribution through secretion of albumin into the circulation.

Altogether, FcRn expression in the liver serves two main purposes: to maintain monomeric IgG and albumin in the circulation and to direct albumin toward the circulation instead of to the bile. Whether removal of small IgG IC from the circulation also relies on FcRn expression by LSEC is unknown.

## Functional Consequence of FcRn Expression in Endothelium

Endothelial cells line the entire vascular system and control the passage of numerous cells and molecules in and out of the circulation, and are one of the major cellular locations where FcRn controls the levels and persistence of IgG and albumin (83). Indeed, FcRn expression by these cells is well-documented in intracellular vesicular compartments (76, 118, 173, 189). As FcRn interactions with its ligands are restricted to intracellular acidic compartments, it is important to note that IgG is thought to be taken up by endothelial cells mainly by pinocytosis (88, 89, 92, 93), while albumin uptake is thought to be facilitated via binding to another albumin receptor, albondin (190). However, to our knowledge, this receptor has neither been sequenced, nor have its functions been recently investigated. While a current study showed localization of internalized albumin in early endosomes and not to lysosomes, which is in line with FcRn mediated rescue from degradation (99), the precise albumin sorting mechanism has not been studied to the same extent as IgG.

It is important to remember that although albumin and IgG are the most abundant proteins in the circulation, two-thirds of total albumin and one-half of IgG reside in the extravascular compartment (191). Whether or not FcRn is involved in the above-mentioned distribution of IgG or albumin, and if so to what extent, is still unknown.

*In vivo* analysis of how FcRn contributes to IgG and albumin biodistribution via endothelial expression is currently lacking, although conditional deletion of murine FcRn in both the vasculature and cells of bone marrow origin (*Tie2*^cre^) results in decreased IgG and albumin levels in the serum (73). The precise vascular location of FcRn remains to be determined, which is complicated by endothelial cell heterogeneity with differences between arteries, veins, large and small vessels, as well as diversity in microvasculature beds from different organs (192). Intracellular trafficking, recycling and transcytosis of IgG and albumin in the endothelia have so far mainly been carried out using cell lines, including human placental endothelial cells (HPEC), human umbilical vein endothelial cells, human dermal microvascular endothelial cells, or mouse SV40-transformed endothelial cells. Using polarized HPEC, it was shown that greater IgG recycling occurred at basolateral cell surfaces, representing the extracellular matrix, compared to the apical cell surfaces, which represents the blood vessel lumen (189). IgG transcytosis was consistently more prevalent in a basolateral to apical direction in HPEC. These results reflect the placental origin of the endothelia used in this study, in which IgGs are transported from maternal to fetal circulation across the endothelial monolayer. More recently, using non-polarized FcRn-transfected human umbilical vein- or dermal microvascular -endothelial cells, the recycling of both IgG and albumin was studied (69, 98, 99). The role of endothelial FcRn in handling IgG IC is not currently known, although as mentioned above LSEC are crucial in elimination of small IC from the circulation.

### The Blood-Brain Barrier

The blood-brain barrier (BBB) restricts access of large molecules to the central nervous system (CNS) by separating the circulation from the CNS. Microscopy studies have shown that FcRn is expressed in brain microvascular endothelium as well as choroid plexus epithelium (193), where it has been suggested to mediate active transport of IgG from the brain into bloodstream (194, 195). In mice, intraperitoneal or intravenous IgG administration resulted in <0.01% of the injected dose to be detected in the brain (196), and at steady state, endocytosed IgG was localized to lysosomes within brain endothelial cells (197). Similarly, albumin is excluded from the CNS (198). In a mouse model of Alzheimer's disease it was shown that FcRn at the BBB was involved in removal of amyloid β-peptide-specific IgG IC (199). In rats, an IgG with improved FcRn affinity was cleared faster from the brain upon intracranial injection, than an IgG with no affinity for FcRn (195). Interestingly upon intra-cerebral injection the efflux of albumin from CNS is slow with an elimination half-life of 10–12 h, whereas IgG efflux is rapid with an elimination half-life of 48 min (194). Still, others have described a more limited role for FcRn in clearance of IgG from the brain (181, 200). Further studies are necessary to fully understand the role of FcRn in the BBB function, and attention should also be given to the choroid plexus epithelium which also expresses FcRn (193).

## Functional Importance of FcRn Expression in Cells of Hematopoietic Origin (HC)

The description of FcRn expression after the neonatal period in adult liver was followed by a demonstration that FcRn is also abundant in cells of bone marrow (BM) origin in adult animals (201). Since then, FcRn presence in humans as well as several animal models including mice, rats and non-human primates has been shown. Thus, FcRn is expressed by monocytes, macrophages (both tissue resident and splenic), neutrophils, DC and B lymphocytes but not by T or natural killer (NK) cells (97, 118, 177, 201–205). However, due to the heterogeneity of these cell subsets, more detailed systemic studies are needed to precisely assess FcRn expression patterns and define species-specific differences. Regardless, the presence of FcRn mainly in antigen presenting cells (APC) indicates that it might provide functional benefits to these cells and directly implicates FcRn in IgG-mediated immune responses.

**Figure 5:**
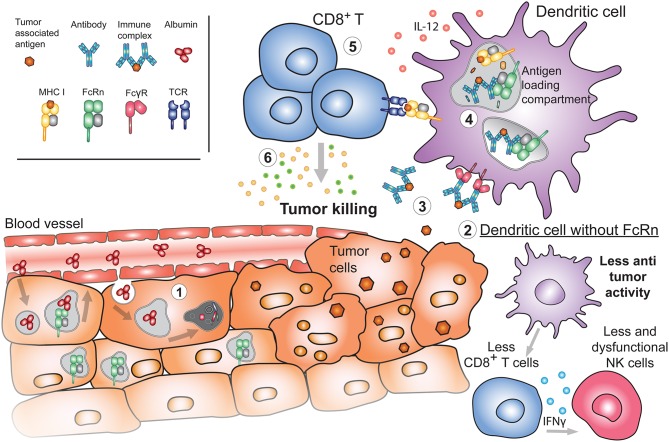
Emerging roles of FcRn in cancer. (1) During the process of oncogenesis, cells can lose or downregulate FcRn expression. In these instances, tumor cells will be unable to recycle albumin upon its internalization. Albumin will instead be degraded, providing nutrients to the tumor and promoting tumor growth. (2) Absence of FcRn in APC may decrease the basal cytotoxic tone (such as IL-12 production) resulting in diminished numbers and function of either NK or CD8^+^ T cells in tissues, generating a tumor-prone environment. (3) Released tumor antigens can be bound by antibodies and internalized by APC through FcγRs. Presence of FcRn in these cells is important for (4) the subsequent sorting and efficient processing of IgG IC in antigen loading compartments where tumor-derived peptides are loaded onto MHC I for cross-presentation to (5) cytotoxic CD8^+^ T cells. Activated tumor specific CD8^+^ T cells will (6) effectively target cancerous cells for destruction.

Overall, immunophenotypic analysis of *Fcgrt*^−/−^ mice revealed subtle decreases in mucosal CD8^+^ T and NK cell frequency (205–207) as well as splenic CD8^+^ T cell frequency when compared to *WT* mice (205) (Figures 2, 5). These cells also displayed functional defects; for instance, CD8^+^ T cells from the large intestinal lamina propria of *Fcgrt*^−/−^ mice secreted less IFN-γ, IL-10, and TNF upon re-stimulation in comparison to *WT* littermates and exhibited inferior cytotoxic activity (206). In the absence of FcRn, NK cell development, maturation and function were impaired as well (205). Furthermore, lung-resident CD103^+^ DC, splenic macrophage and neutrophil subsets were increased (205, 207). As FcRn is not expressed by NK and CD8^+^ T cells, the observed defects were possibly associated with abnormal cytokine response of myeloid cells which affected the function of several other cell subsets in trans. Indeed, a similar defect in CD8^+^ T cells was present when FcRn was conditionally deleted in CD11c^+^ cells (*Itga*x^*cre*^*Fcgr*t^*fl*/*fl*^) (206). In addition, *Fcgr*t^−/−^ DC exhibited decreased expression of IFN-γ, IL-12p35, T-bet, and TNF, which are all necessary for effective cytotoxic T cell-mediated immunity (206).

Further, it is noteworthy to contemplate the possibility that FcRn, a non-classical MHC-I molecule, may interact with one of the NK cell receptors that are acknowledged to bind classical and non-classical MHC-I family members (208). More importantly, in view of these results, studies of decidual NK cells, that are critical for the uterine spiral artery remodeling (209), become essential, given the documented importance of FcRn during fetal development.

Outside of the intrinsic cellular consequences, up to now the major attention of the scientific community on the function of FcRn in *sensu stricto* immunity has focused on how it affects the half-life and biodistribution of IgG. Thus, the important contribution of the hematopoietic FcRn compartment to immunity *per se* was unanticipated. Nonetheless, this was demonstrated in a series of studies with BM chimeric mice whereby FcRn deficient, *WT* or *FCGRT*^TG^ mice were used as donors and recipients interchangeably (*Fcgrt*^−/−^ BM Donors → *WT* or *FCGRT*^TG^ Recipients; *WT* or *FCGRT*^TG^ BM Donors → *Fcgrt*^−/−^ Recipients). Results of these studies showed that BM derived cells, in addition to vascular endothelial, epithelial, stromal and parenchymal cells, are necessary to extend the half-life of monomeric IgG in the circulation (97, 177, 210). Data from conditional deleted mice, where FcRn was absent either in vascular and hematopoietic (*Tie2*^cre^*Fcgrt*^fl/fl^) or CD11c (*Itgax*^cre^*Fcgrt*^fl/fl^) compartment corroborated these findings (54, 73). More recently, Challa and colleagues have described the effects of conditional FcRn deletion in macrophages or B cells and DC (211). They showed that the absence of FcRn in macrophages (*Lys*M^*cre*^*Fcgrt*^fl/fl^), but not the latter cells (*CD19*^*cre*^*Fcgrt*^fl/fl^), results in excessive IgG degradation as IgG half-life and circulating levels were drastically reduced as compared to *WT* animals. Interestingly, in some instances the Cre recombinase activity is not exclusively operating in DCs (*Itgax*^cre^) or macrophages (*Lys*M^*cre*^), and varies from tissue to tissue which can affect the level of deletion and perhaps affect other HC cells (212). Nonetheless, it is clear that FcRn deletion in cells of bone marrow origin decreases the levels and half-life of circulating IgG (54).

Similar types of experiments illustrated the contribution of HC to albumin homeostasis. Thus, on the one hand *Fcgrt*^−/−^ BM chimeras (*Fcgrt*^−/−^ BM Donors → *WT* Recipients) displayed lower circulating albumin levels when compared to *WT* mice (210). Still these levels were significantly higher than observed in complete *Fcgrt*^−/−^ animals. On the other hand, reconstitution of *Fcgrt*^−/−^ mice with *WT* BM (*WT* BM Donors → *Fcgrt*^−/−^ Recipients) only partially restored circulating albumin levels (210). Furthermore, while conditional deletion of FcRn in vascular and hematopoietic (*Tie2*^cre^) as well as macrophage (*Lys*M^*cre*^) compartments resulted in about 2-fold lower albumin levels in the serum (73, 211), no changes were observed when FcRn was deleted in the CD11c compartment (*Itgax*^cre^) (54). Thus, the deletion of FcRn in DC or other cells of HC origin does not affect circulating albumin levels to the same degree as IgG, and suggests that a considerable fraction of albumin recycling occurs primarily in non-HC compartments, although compensatory effects may also be at play.

Besides being a major site of monomeric IgG and albumin protection from degradation, HC expressing FcRn play an important role in immune responses to IgG bound antigens in form of IC. Indeed, experiments with FcRn^−/−^ BM chimeras (*Fcgrt*^−/−^ BM Donors → *Fcgrt*^+/+^ Recipients) injected with small IgG IC have shown that the absence of FcRn in HC dramatically reduced the persistence of these complexes in the circulation (97). Larger IC formed by monoclonal anti-NIP IgG antibody and NIP-conjugated antigens (consisting of 15 NIP molecules per antigen) were cleared faster than small IgG IC but still were protected in an FcRn-dependent manner in HC (97). This is consistent with the observation in epithelial cell lines, that monomeric or small IgG IC are recycled while large IgG IC are diverted to late endosomes and/or lysosomes where they are retained for extended periods of time (96, 97, 213). In addition, FcRn cross-linking by IgG IC induces a signaling cascade that is associated with secretion of IL-12 and is overall skewed toward T helper 1 and T cytotoxic responses (206, 214). Given that low levels of circulating IC have been detected even in healthy individuals and animals (215–217), a basal amount of these IC might provide, via interaction with FcRn, a basal cytokine tone essential for HC homeostasis and explain the immunophenotype of *Fcgr*t^−/−^ mice described above that include decreased inflammatory tone of HC as well as diminished NK and CD8^+^ T cells functions (206). More importantly, in response to variable amounts of IgG IC, FcRn in HC would affect the outcome of an immune response.

Such responses are buttressed by the HC expression of low-affinity classical FcγR (FcγRIIa/b/c, FcγRIIIa/b) (Box 1) (10), which are mostly present at the cell surface, and interact with IgG IC rather than monomeric IgG, at neutral pH (218). Therefore, instead of pinocytosis or unspecific fluid phase endocytosis, HC are able to efficiently internalize IgG IC via receptor-mediated endocytosis which triggers particular signaling pathways (219), and may affect subsequent intracellular FcRn encounters with these FcγR-IgG IC complexes. It is well-recognized that FcγR triggered immune responses to IgG IC potentiate the processing of antigen contained within IC (220, 221). These can culminate either in MHC-I cross-presentation or MHC-II presentation to CD8^+^ and CD4^+^ T cells, respectively; however the degree and interdependence of FcRn in these processes are still emerging. Thus, mouse APC or human monocyte derived DC exposed to IgG^WT^ IC, but not to the FcRn non-binding variant IgG^IHH^ IC, induce greater CD4^+^ T cell proliferation (97). CD8^+^ T cell responses to cross-presented antigen contained within IgG IC are similarly dependent on FcRn, with one main difference. While the DC population that mediates cross-presentation of soluble antigens (CD8^+^CD11b^−^ DC) in mice exhibits little dependence on FcRn, the CD8^−^CD11b^+^ DC population relies significantly on FcRn to efficiently cross-present antigen contained within IgG IC and stimulate CD8^+^ T cells both *in vitro* and *vivo* (214). In this instance, FcRn was important for movement of IgG IC into phagosomal compartments conducive to cross-presentation in addition to preventing their fast and excessive degradation in association with intracellular retention (214). In neutrophils, FcRn enhanced phagocytosis of IgG-opsonized bacteria and their delivery into phagolysosomes as compared to *Fcgrt*^−/−^ cells (202). Interestingly, neutrophils treated with IgG IC that retained normal binding to FcγR but were unable to bind FcRn displayed reduced phagocytosis (202), suggesting that in some HC subsets classical FcγR and FcRn might function in parallel and not sequentially. The mechanisms underlying these observations need to be further established.

Dating back to Paul Elrlich (222) and F.W. Rogers Brambell (137), the initial impetus to study and understand passive immunity was the protection of the offspring from infection. Nevertheless, accumulating evidence illustrates that FcRn participates not only in the transfer of protective immunity but tolerance as well (223). Studies of murine materno-fetal and neonatal IgG transport clearly illustrate that FcRn plays an important role in induction of tolerance, however whether this effect is dependent on FcRn within HC was unknown until now (131). Using Fc-fused hemagglutinin and T cell receptor TG mice specific for hemagglutinin, Gupta et al. have illustrated that FcRn-dependent transplacental transport of Fc-hemagglutinin induced tolerance via antigen-specific regulatory T (T_reg_) cells (156). In a similar type of experiment, the administration of Fc fused preproinsulin to pregnant mice resulted in efficient passage of these chimeric proteins to fetuses and prevented development of autoimmune diabetes. More specifically, Fc-preproinsulin was carried to the thymus by migratory DCs and provided support for the emergence of antigen-specific thymic-derived CD4^+^ T_reg_ cells as well as induced development of impaired cytotoxic CD8^+^ T cells (157). Furthermore, in an allergic airway disease model, it was shown that post-partum exposure of lactating female mice to airborne antigens led to decreased airway hyper-reactivity only in breastfed offspring, which was associated with the presence of TGF-β as well as IgG IC in the milk (133). Thus, it was the FcRn-mediated IgG IC transfer to the newborn that induced antigen-specific Foxp3^+^ T_reg_ cells (132). Still, these studies mostly emphasized FcRn as a transplacental delivery receptor of IgG IC that permitted antigen delivery to APC, without specifically investigating FcRn's role within these cells as a mediator of tolerance. More recently, in an epicutaneous sensitization model of pregnant mice, the offspring of allergic mothers became tolerant to a food allergen challenge, whereas those of non-allergic mothers developed signs of systemic anaphylaxis (134). In protected offspring, FcRn was responsible for the transfer of maternal IgG IC from breast milk to neonates, induction of allergen-specific Foxp3^+^ T_reg_ cells and long-term reduction in anaphylaxis to food allergen that persisted long after maternal-derived antibodies had disappeared. Most importantly, conditional deletion of FcRn within the APC population (*Itga*x^*cre*^*Fcgr*t^*fl*/*fl*^) in offspring of OVA-sensitized mothers failed to exhibit tolerance to food allergy (134). These findings illustrate that fetal and neonatal HC expressing FcRn actively promulgate tolerance to antigens comprised within maternally acquired IgG IC (Figure 3). Critically, such processes extend beyond the half-life of transferred IgG and can potentially revise our concepts of passive immunity.

## Emerging Role of FcRn in Cancer

Given these observations, it is not surprising that FcRn expression in the HC promulgates antitumor activity as illustrated by the increased susceptibility of *Fcgrt*^−/−^ mice to tumor development in models of colorectal cancer and lung metastasis (205, 206) (Figure 5). For instance, *Fcgrt*^−/−^ mice exposed to the chronic carcinogen, azoxymethane and dextran sodium sulfate displayed deficient frequency and function of tissue and adjacent CD8^+^ T cells, which resulted in inability to control tumor growth in comparison to their *WT* littermates. These defects in CD8^+^ T cell numbers were dependent on the FcRn expressing CD8^−^CD11b^+^ DC fraction, as adoptive transfer of *WT* DC conferred protection to *Fcgrt*^−/−^ recipients. In addition, DC from *Fcgr*t^−/−^ mice were deficient in the production of cytokines propagating cytotoxic T cell responses as mentioned above (206). Furthermore, a recent report described downregulation of FcRn expression in individuals with non-small cell lung carcinoma, which was associated with poor patient survival (224), consistent with other studies in colorectal cancer (206). More specifically, FcRn was significantly less abundant in lung tumor than non-cancerous tissue. Conversely, high FcRn expression in both cancerous and non-cancerous cells such as macrophages and DC was associated with a favorable prognosis (224).

Supporting a central role of FcRn in tumor biology is another observation reported by the Ward laboratory, but in contrast to the above it pertains to the FcRn-albumin interaction (225). The active internalization of albumin by tumor cells was recognized long before its interaction with FcRn was discovered (226). Swiercz et al. illustrated that numerous cell lines derived from breast and prostate tumors were characterized by greatly reduced FcRn expression levels. This allowed them to accumulate more albumin within cells due to reduced FcRn dependent recycling (225) (Figure 5). Albumin was instead diverted to and degraded in lysosomes, serving as a nutrition source for the tumor. In mouse xenograft studies, inoculation of FcRn expressing tumors resulted in more restricted growth as compared to FcRn deficient tumors which displayed accelerated tumor expansion (225). In line with this it was also recently reported that albumin conjugated to the drug doxorubicin showed better tumor inhibition efficacy in pancreatic cancer when FcRn expression was reduced. This was caused by reduced FcRn recycling, leading to increased albumin-drug catabolism (227). Overall, these results reveal that FcRn in HC and non-HC is involved in extrinsic and intrinsic control of tumor growth and that modulating FcRn function might be exploited as anti-tumor therapy.

## FcRn-Based Therapeutics

Our growing understanding of FcRn's molecular structure, ligand binding properties, patterns of expression and biological functions have led to the development of therapies that aim to either exploit FcRn binding or to block it. Therefore, FcRn-based therapeutics can be subdivided in three general groups: targeted delivery, half-life extension or enhanced clearance approaches.

### Targeting FcRn for Delivery of Therapeutics

There has been a great desire for enabling non-invasive delivery of therapeutics across mucosal surfaces. In addition, most communicable infections are initiated at mucosal sites, and the ensuing protective immunity involves activation of local immune cells. The role of FcRn at these locales in shuttling its ligands across the protective epithelial cell layer has thus led to the emergence of therapeutics aimed at enhancing transport of biologics across mucosal surfaces, to improve drug absorption or distribution. Indeed, fusions to IgG Fc or albumin have proven effective in pulmonary, oral, genital, and *in utero* delivery of therapeutics or vaccines.

Ye et al. showed that targeting FcRn is an effective method for transepithelial delivery of a vaccine consisting of a herpes simplex virus type-2 glycoprotein D-Fc fusion. Intranasal immunization of mice using such a construct induced efficient mucosal and systemic antibody, B and T cell immune responses, and procured stable protection for at least 6 months after vaccination (228). In another study, intranasal immunization with Fc fused human immunodeficiency virus gag protein was found to induce local and systemic immunity, as well as protection at distal mucosal sites upon vaginal challenge with a recombinant vaccinia virus expressing the human immunodeficiency virus gag protein (229). Furthermore, Pridgen et al. used Fc conjugated nanoparticles to target FcRn in the intestine for delivery of insulin across the epithelium, where it showed efficient uptake and distribution to various tissues. This delivery was dependent on FcRn as demonstrated by administration of insulin-loaded Fc nanoparticles to *WT* (33) or *Fcgrt*^−/−^ mice where only the *WT* mice exhibited significant hypoglycemia (230).

Unsurprisingly, FcRn targeted therapies hold promise for fetal and neonate medicine. In one murine study, the ability of FcRn to transport IgG across the placenta was exploited to deliver an enzyme to treat lysosomal storage disease *in utero*. This was achieved through the administration of the enzyme beta-glucuronidase-Fc fusion protein to pregnant mothers which resulted in delivery of active enzyme to the fetal circulation and alleviated clinical findings associated with fetal beta-glucuronidase deficiency (231). Successful Fc-associated cargo delivery to the fetus was also recently shown for preproinsulin- and factor VIII (FVIII)-Fc fusion proteins (156, 157).

In non-human primates, FcRn expressed in the lung has been shown to enable delivery of erythropoietin (Epo) when fused to Fc and provided a distribution similar to that of Epo monomer alone delivered subcutaneously (33). The same Fc fusion Epo molecule could also be used for delivery by inhalation in humans resulting in the presence of the fusion protein constructs in serum and increase in circulating reticulocytes (232). In addition, Fc-fusion proteins of interferon-α, interferon-β and follicle-stimulating hormone can be delivered in an FcRn dependent manner via the pulmonary route (33, 232–234).

So far, the demonstration that albumin fusions can be delivered across epithelium via an FcRn/β_2_m-dependent mechanism has not been established, even though albumin can be transcytosed by polarized FcRn expressing MDCK II cells in the same way as IgG (54). Albumin is present in large quantities at mucosal surfaces, similar to IgG, and in extravascular spaces (235). Further, albumin is known to be highly water-soluble and stable, and challenges related to mucosal delivery of protein-based drug formulations such as low pH, protein instability, and poor absorption may support albumin fusions as an advantageous delivery platform, as reviewed in Sleep (236). Importantly, compared to Fc as a delivery unit, albumin does not bind to classical FcγR and thus may lower the risk for unwanted immune activation. Liu et al. took advantage of albumin for efficient delivery of vaccine antigens into lymph nodes. This was achieved through attaching a fatty acid to the antigen, which bound to albumin in the circulation and further lead to lymph node accumulation (237). Interestingly, the fatty acid consisted of C18 diacyl lipid tails which bind albumin and block its interaction with FcRn (45, 54), suggesting that lymph node accumulation might have resulted from inability to engage FcRn-mediated recycling. These examples illustrate that targeting of FcRn is an efficient approach to non-invasive delivery of therapeutics and vaccines.

### Half-Life Modification

Given the expanding use of monoclonal antibodies (mAb) as treatment in a range of human ailments including chronic inflammation, infections, cancer, autoimmune diseases, cardiovascular diseases and transplantation medicine, FcRn has emerged as major modifier of mAb efficacy (238, 239). This is directly related to the persistence of the therapeutic antibody in the bloodstream, which in turn can increase localization to the target site. To ensure long circulatory half-life of IgG, pH dependent binding and FcRn dependent recycling are crucial. Importantly, limited binding at neutral pH is required for proper release of IgG from cells and increasing the mAb affinity to FcRn at acidic pH correlates with half-life extension. Thus, IgG Fc engineering to optimize pH dependent binding to FcRn has been explored to tailor pharmacokinetics and increase mAb half-life (240–242). For example, the MST mutations (Met252Tyr/Ser254Thr/Thr256Glu) have enabled up to 5-fold increased persistence of IgG in humans and monkeys (240). In Phase II clinical trials the IgG^MST^ variant demonstrated half-lives of 80–120 days (242). Similarly, MN (Met428Leu/Asn434Ser) mutations, that are adjacent to the critical FcRn binding site on IgG Fc, show promise in extending IgG half-life for therapeutic antibodies (242).

Antibody engineering approaches have also been developed for more rapid degradation of target molecules, for instance toxins or inflammatory cytokines. Examples of such systems are acid-switched or calcium switched antibodies as reviewed in (243), that dissociate from their antigen at acidic pH or at lower calcium concentrations which are found in endosomal vesicles. Such antibodies will therefore bind to their target in the bloodstream and be taken up by cells. Once within the endosomal compartments, the antigen will disengage from the antibody ensuring intracellular degradation of the antigen, whereas the antibody would be protected from degradation by FcRn and recycled. In this way, the antigen circulatory half-life is limited, whereas the long half-life of the therapeutic antibody is preserved rendering it more effective even at sub-stoichiometric levels.

The ability of FcRn to prolong the half-life of its two ligands can also be exploited to extend half-lives of therapeutics by fusing a short-lived protein of interest to the Fc part of IgG or albumin. The first such fusion approved for clinical use was Etanercept (Enbrel®), which consists of the TNF receptor extracellular domain fused to the Fc part of human IgG1 (244). Etanercept competes for TNFα and TNFβ with TNF receptor and is used for treatment of rheumatoid arthritis and other forms of autoimmunity, including inflammatory bowel disease (245).

The Fc-fusion technology has also resulted in new therapeutics for treatment of hemophilia. Hemophilia A and B are X-linked bleeding disorders resulting from deficiencies of coagulation factor VIII (FVIII) and factor IX (FIX), respectively (246). Until recently, treatment required frequent injections of these factors to prevent spontaneous bleeding. A recombinant FVIII (rFVIII) fused to the Fc fragment of IgG1 (Eloctate®) was approved for clinical use in 2014. FVIII-Fc is a heterodimer that consists of one Fc chain fused to FVIII, while the other Fc is unfused, the so-called monomeric Fc-fusion (33, 247). Monomeric rFVIII-Fc allowed for less frequent administration, occurring every 4–7 days instead of every 2–3 days for rFVIII alone (248). Monomeric rFIX fused to IgG1 Fc (Alprolix®) was also approved for clinical use in 2014 and provides 3–5-fold longer half-life when compared to the rFIX alone (249).

Albutrepenonacog alfa (Idelvion®) is a fusion protein linking rFIX with albumin. A cleavable linker between rFIX and albumin is derived from the endogenous activation peptide in native FIX (250). Factor IX fused to albumin was approved for clinical use in March of 2016; this drug reduces the frequency of injections to once every 2 weeks, instead of the 2 weekly injections for rFIX alone (251–253). It was recently also shown that the albumin rFIX fusion localizes to Rab11a positive FcRn endosomes which supports the role of FcRn in promoting extended serum half-life (254). Another albumin fusion product currently approved for clinical use in the treatment of type II diabetes is Albiglutide (Eperzan®/Tanzeum®) (255). It consists of fusion of glucagon-like peptide-1, which stimulates insulin secretion by pancreatic β cells, to albumin (256). Albiglutide has a half-life of 5 days in humans (compared to minutes for unfused glucagon-like peptide-1) which allows for weekly injection regimens (255, 257). Several therapeutics based on albumin are under development or in clinical trials, and show promising results, as reviewed in (53, 236). Like IgG Fc region engineering, albumin variants with improved binding to FcRn and increased half-life are emerging. One such modified albumin, which is distinguished by a Lys-to-Pro substitution at position 573 of DIIIB, has 12-fold increased affinity for FcRn, which resulted in a significant increase in circulatory half-life in cynomolgus monkeys (258).

One major obstacle of replacement therapies is the emergence of immune responses to the therapeutic recombinant proteins in the form of neutralizing antibodies, reviewed in (259). This is exemplified by hemophilia A and B, because 40 and 4% of patients receiving rFVIII or rFIX, respectively, develop antibodies against the factor (260–262). Emerging clinical and experimental data suggest that this may less likely be the case with the Fc fusions, as the rFVIII-Fc and rFIX-Fc appear less immunogenic than the unconjugated recombinant factors alone (263–265). This is believed to occur via induction of tolerance through yet uncharacterized FcRn-dependent and -independent mechanisms (266, 267). A similar absence of immunogenicity has been described for the albumin-FIX fusion (253). Consequently, IgG Fc-, and perhaps eventually, albumin-fusion therapies might possess another unanticipated advantage by being more tolerogenic, in addition to mediating extended half-life.

### Enhanced Clearance of IgG and Albumin

IgG and albumin permeate the host body and are normally innocuous. Yet in particular instances they may be harmful. This is extremely well-documented in certain autoimmune diseases, in which pathogenic self-reactive IgG antibodies play central roles (268–270). Decreasing the circulating levels of these auto-antibodies could therefore be beneficial (271, 272), and as such, the blockade of FcRn has been predicted to alleviate IgG-mediated autoimmune diseases (21, 268, 273, 274).

Several strategies have been used, including engineering antibodies with Fc regions that bind at neutral and acidic pH, anti-FcRn antibodies that block the IgG binding site, and FcRn-inhibitory peptides and small proteins (273, 275–283). Further, efficient FcRn blocking requires superior, pH independent binding to the receptor. Currently four such FcRn blocking molecules have entered clinical trials: Efgartigimod, M281, Rozanolixizumab, SYNT001, and IMVT-1401.

Efgartigimod is a human IgG1 Fc fragment that contains a constellation of “MST/HN” mutations (Met252Tyr/Ser254Thr/Thr256Glu/His433Lys/Asn434Phe) resulting in pH independent (${\text{K}}_{\text{D}}^{\text{pH}6}$ = 14.2 nM, ${\text{K}}_{\text{D}}^{\text{pH}7.4}$ = 320 nM), high affinity binding to hFcRn (282). Thus, upon engaging FcRn, Efgartigimod, occupies the receptor and prevents its interaction with and salvage of circulating IgGs. As this therapeutic agent possesses relatively low affinity for human FcRn at neutral pH, it is also to some degree recycled. In mice, this strategy was shown to enhance IgG clearance and to significantly reduce pathology in K/BxN arthritis and experimental autoimmune encephalomyelitis models (280, 284). In a phase I clinical trial, Efgartigimod treatment produced a rapid reduction of circulating IgG levels clearly demonstrating the effectiveness of this approach (277). At the highest administered dose of 50 mg/kg, Efgartigimod reduced all subclasses of IgG levels by approximately 50%, and multiple administration regimens (every 4 days at 10 mg/kg or 7 days at 25 mg/kg) reduced IgG levels by up to 75% (277). These effects were long lasting as antibody levels did not return to their baseline for 8 weeks post-administration. A phase II study was also recently completed where myasthenia gravis patients treated with Efgartigimod showed rapid decrease of total IgG and autoantibodies (285). Interestingly, the MST/HN mutations of IgG Fc was at the core of another approach to specifically deplete pathogenic antibodies, but it has not yet progressed to clinical trials. This strategy consists on fusing MST/HN Fc with the antigen to which the pathogenic antibody binds (286). While the antigen portion binds and traps the autoimmune antibody, the Fc portion strongly binds to FcRn, and directs the complex for rapid degradation.

Contrary to Efgartigimod, M281 is an human anti-FcRn IgG1 antibody that binds and blocks the IgG Fc binding site on FcRn (279). M281 has picomolar affinity for FcRn at both acidic (${\text{K}}_{\text{D}}^{\text{pH}6}$ = 43.5 pM) and neutral (${\text{K}}_{\text{D}}^{\text{pH}7.4}$ = 28.7 pM) pH. A single administration of M281 at 60 mg/kg reduced circulating IgG levels within 2 weeks by approximately 80% from baseline. At this dose, a 20% decline from baseline was still seen 2 months after administration (279).

Another mAb designed to block IgG binding site on FcRn, Rozanolixizumab, is an IgG4P isotype that reduced IgG levels by ~45% in humans when administered at 7 mg/kg dose (278). Although other classes of circulating Abs were not affected by Rozanolixizumab and M281 administration, a slight decrease in albumin levels was observed, possibly caused by steric hindrance at the FcRn-albumin interaction site by the bound therapeutic antibody (278, 279). Future clinical trials will show if and to which extent the depletion of circulating IgG can also affect susceptibility to infectious diseases.

Aside from blocking the IgG-FcRn interactions, there is also premise that hindering albumin binding to FcRn might also be beneficial, although the evidence for a pathological role of albumin is more ambiguous. For instance, abnormal levels of glycated albumin, observed in diabetic patients, have been associated with disease pathogenesis and tissue inflammation (287–289). Further, albumin as a carrier protein associates with many hormones, ions, metabolites and drugs, and can extend their *in vivo* half-life. Some of these molecules might be harmful at high dose, and their binding to albumin may prolong or maintain their levels in the toxic range. Acetaminophen (APAP), is a widespread analgesic that binds to albumin, and its overdose results in severe liver toxicity. We have shown that depleting albumin either via genetic deletion of FcRn or FcRn inhibition via delivery of antibodies or peptides that block the FcRn-albumin interaction, decreased APAP-mediated toxicity in mice. Although the precise mechanism of protection was not identified, it correlated with increased transport of APAP-loaded albumin into the bile and the accumulation of albumin within the hepatocyte that enhanced the intracellular albumin-mediated scavenging of reactive oxygen species (54). The therapeutic utility in blocking or enhancing albumin-FcRn interactions is less well-explored compared to IgG, largely due to inadequate understanding of albumin biology and pathology, but these studies demonstrate some potential for the albumin-docking site on FcRn as a target for future basic and translational research.

## FcRn Is a Receptor for Echoviruses

Most recently, FcRn has been identified as a receptor critical for infection with Echoviruses (290, 291) (Box 5), which are the leading cause of viral encephalitis and meningitis in children (294). The lack of FcRn, was thus shown to render the cells resistant to Echovirus infection while expression of hFcRn^TG^ in mice enhanced viral infection (290). Another group illustrated binding of FcRn at neutral and acidic pH to Echovirus virions (291). Importantly, given that viral uncoating and genome release occur in acidic environment, typical of FcRn-rich endosomes, it directly implicated FcRn as Echovirus uncoating receptor. Further studies are necessary to define the precise role of this unusual Fc receptor in echovirus pathogenesis.

Box 5 **Echoviruses.**

Echoviruses belong to the species *Enterovirus B*, the genus *Enterovirus* of the *Picornaviridae* family. They make up the largest Enterovirus subgroup, consisting of 29 serotypes. Echoviruses are common human pathogens causing a range of illnesses such as febrile illness, but also potentially fatal conditions such as aseptic meningitis, encephalitis, paralysis and myocarditis (292, 293).

## Emergence of FcRn Functions During Vertebrate Evolution

Lastly, it is interesting to reflect on the emergence of a receptor with an MHC-I fold, that instead of peptide presentation, binds not only IgG but also albumin in a pH-dependent fashion, and Echoviruses in a pH-independent manner. Investigation of materno-fetal transfer of antibodies have shown that aside from mammals, birds and some reptiles can transport circulating IgY, an IgG ortholog, from the mother to the yolk (295–298). In mammals and marsupials, whole genome sequence analysis of non-classical MHC-I molecules clearly identified the presence of a gene encoding the heavy chain of FcRn, while a pseudogene was detected in monotremes (299). More recently, Dijkstra et al. studying the conservation of MHC-I molecules sequence motifs, estimated that the *FCGRT* separated from the classical MHC-I lineage before the separation of monotremes and mammals around 163 million years ago (300), sometime after the divergence of amphibians and amniotes around 330 million years ago (301). Although the authors further propose that FcRn coevolved together with lactation in early mammals, this would only explain unidirectional IgG and albumin transfer into the milk and does not take into account the emergence of IgG or albumin recycling via FcRn. Further, in light of recent findings with Echoviruses, possible evolutionary pressure shaped by viral infection should also be taken into consideration. Though extremely intriguing, the study of the emergence of FcRn is still in its infancy.

## Conclusion

FcRn controls the fate of two very distinct proteins, IgG and albumin, through a highly similar mode of binding. Despite this, FcRn's relationship with these two ligands likely involves poorly understood cooperation with other cell surface proteins and activity in different cell types that allow FcRn to manage these functionally unique proteins. Just as importantly, it is increasingly clear that FcRn functions throughout life within numerous cell types and with functional implications that are expanding into completely new areas of biology beyond what was originally envisioned. As such, the knowledge that has accumulated over the past 50 years since FcRn was imagined as a potential cellular receptor is now finally being co-opted for many exciting therapeutic purposes and in numerous areas involving drug delivery, antibody engineering, autoimmunity, cancer, and undoubtably others. It is therefore very clear that FcRn mediates much more interesting biology than its name implies.

## Author Contributions

MP and KS wrote the manuscript and prepared the figures. JH, JA, IS, and RB wrote and edited the manuscript.

### Conflict of Interest Statement

RB and IS had equity interests in Syntimmune, Inc., a company developing therapeutic agents to target FcRn. Syntimmune, Inc. is now a wholly-owned subsidiary of Alexion Pharmaceuticals, Inc., following its acquisition by Alexion. The remaining authors declare that the research was conducted in the absence of any commercial or financial relationships that could be construed as a potential conflict of interest.
